# Supplementary material for: Community deployment of a synthetic pheromone of the sand fly Lutzomyia longipalpis co-located with insecticide reduces vector abundance in treated and neighbouring untreated houses: Implications for control of Leishmania infantum
Source: PLoS Negl Trop Dis. 2021 Feb 3;15(2):e0009080. doi: 10.1371/journal.pntd.0009080 (PMC7886189; doi:10.1371/journal.pntd.0009080)
Supplement: S1 Table — (DOCX) [file pntd.0009080.s001.docx]

| Study block | True Control arm | Untreated arm | Pheromone + insecticide arm | Total households |
| --- | --- | --- | --- | --- |
| **pre-intervention** | |  |  |  |
| round 1 (~106 days pre-intervention) | | |  |  |
| 1 | 12 | 13 | 14 | 39 |
| 2 | 17 | 18 | 16 | 51 |
| 3 | 13 | 12 | 13 | 38 |
| 4 | 12 | 12 | 12 | 36 |
| 5 | 15 | 15 | 15 | 45 |
| 6 | 12 | 14 | 15 | 41 |
| 7 | 12 | 11 | 12 | 35 |
| 8 | 12 | 11 | 12 | 35 |
| Subtotal trap nights | 105 | 106 | 109 | 320 |
|  |  |  |  |  |
| **post-intervention follow-up** | | |  |  |
| round 2 (~30 days post-intervention) | | |  |  |
| 1 | 11 | 13 | 12 | 36 |
| 2 | 17 | 17 | 15 | 49 |
| 3 | 12 | 11 | 11 | 34 |
| 4 | 12 | 12 | 10 | 34 |
| 5 | 11 | 14 | 12 | 37 |
| 6 | 9 | 13 | 12 | 34 |
| 7 | 11 | 11 | 11 | 33 |
| 8 | 12 | 11 | 9 | 32 |
| Subtotal trap nights | 95 | 102 | 92 | 289 |
|  |  |  |  |  |
| round 3 (~60 days post-intervention) | | |  |  |
| 1 | 11 | 13 | 11 | 35 |
| 2 | 15 | 17 | 15 | 47 |
| 3 | 12 | 11 | 10 | 33 |
| 4 | 12 | 12 | 10 | 34 |
| 5 | 11 | 14 | 10 | 35 |
| 6 | 9 | 13 | 12 | 34 |
| 7 | 11 | 11 | 11 | 33 |
| 8 | 12 | 11 | 8 | 31 |
| Subtotal trap nights | 93 | 102 | 87 | 282 |
|  |  |  |  |  |
| round 4 (~90 days post-intervention) | | |  |  |
| 1 | 5 | 7 | 6 | 18 |
| 5 | 7 | 5 | 7 | 19 |
| 6 | 7 | 12 | 10 | 29 |
| Subtotal trap nights | 19 | 24 | 23 | 66 |
|  |  |  |  |  |
| **Total trap nights** | **312** | **334** | **311** | **957** |
