# Supplementary material for: Community deployment of a synthetic pheromone of the sand fly Lutzomyia longipalpis co-located with insecticide reduces vector abundance in treated and neighbouring untreated houses: Implications for control of Leishmania infantum
Source: PLoS Negl Trop Dis. 2021 Feb 3;15(2):e0009080. doi: 10.1371/journal.pntd.0009080 (PMC7886189; doi:10.1371/journal.pntd.0009080)
Supplement: S2 Table — (DOCX) [file pntd.0009080.s002.docx]

| Sentinel house trapping round | True Control arm | Pheromone + insecticide arm | Total households |
| --- | --- | --- | --- |
| pre-intervention | |  |  |
| 1 | 17 | 23 | 40 |
| post-intervention | |  |  |
| 2 | 17 | 23 | 41 |
| 3 | 16 | 23 | 40 |
| 4 | 16 | 23 | 40 |
| 5 | 15 | 23 | 39 |
| 6 | 14 | 23 | 38 |
| 7 | 13 | 22 | 36 |
| 8 | 12 | 17 | 30 |
| 9 | 9 | 8 | 18 |
| Total trap nights | 129 | 185 | 322 |
