# Supplementary material for: Community deployment of a synthetic pheromone of the sand fly Lutzomyia longipalpis co-located with insecticide reduces vector abundance in treated and neighbouring untreated houses: Implications for control of Leishmania infantum
Source: PLoS Negl Trop Dis. 2021 Feb 3;15(2):e0009080. doi: 10.1371/journal.pntd.0009080 (PMC7886189; doi:10.1371/journal.pntd.0009080)
Supplement: S3 Table — Houses were sampled on one trap night per pheromone dose. (DOCX) [file pntd.0009080.s003.docx]

| Study block | Number households | Total trap nights |
| --- | --- | --- |
| 1 | 36 | 72 |
| 2 | 49 | 98 |
| 3 | 35 | 70 |
| 4 | 34 | 68 |
| 5 | 39 | 78 |
| 6 | 36 | 72 |
| 7 | 33 | 66 |
| 8 | 33 | 66 |
| Totals | 295 | 590 |
