# Supplementary material for: Community deployment of a synthetic pheromone of the sand fly Lutzomyia longipalpis co-located with insecticide reduces vector abundance in treated and neighbouring untreated houses: Implications for control of Leishmania infantum
Source: PLoS Negl Trop Dis. 2021 Feb 3;15(2):e0009080. doi: 10.1371/journal.pntd.0009080 (PMC7886189; doi:10.1371/journal.pntd.0009080)
Supplement: S4 Table — (DOCX) [file pntd.0009080.s004.docx]

| GV district | Study block | Median distance (m) | IQR | min | max |
| --- | --- | --- | --- | --- | --- |
| Jardim do Trevo | 1 | 14.8 | 10.0-22.7 | 6.4 | 34.8 |
|  | 2 | 13.5 | 11.7-18.2 | 2.2 | 26.1 |
|  | 3 | 15.2 | 11.5-19.9 | 8.2 | 39.0 |
|  | 4 | 18.1 | 17.4-19.2 | 5.7 | 30.4 |
| Santa Rita | 5 | 17.2 | 13.7-21.2 | 4.3 | 40.9 |
|  | 6 | 18.1 | 14.8-25.4 | 3.7 | 45.2 |
|  | 7 | 18.1 | 14.6-23.3 | 6.3 | 37.9 |
|  | 8 | 13.5 | 10.3-24.0 | 3.9 | 30.9 |
