# Supplementary material for: Community deployment of a synthetic pheromone of the sand fly Lutzomyia longipalpis co-located with insecticide reduces vector abundance in treated and neighbouring untreated houses: Implications for control of Leishmania infantum
Source: PLoS Negl Trop Dis. 2021 Feb 3;15(2):e0009080. doi: 10.1371/journal.pntd.0009080 (PMC7886189; doi:10.1371/journal.pntd.0009080)
Supplement: S5 Table — (DOCX) [file pntd.0009080.s005.docx]

| Variable | Category characteristics (% of trap nights) | N trap nights in analysis | LRT χ^2^ [df] (p=)^1^ |
| --- | --- | --- | --- |
| CDC location (cdcloc3) | 1.open ground (18%)  2.amongst vegetation (66%),  3.under outside shelter (4%),  4.outside shelter amongst vegetation (12%) | 932 | 3.47 [3] (0.324) |
| Pheromone dispenser location (ODRS locale) | 1.outside wall (93%)  2.animal shelter (6%) | 636 | 0.03 [1] (0.869) |
| Insecticide sprayed substrate (substr4) | 1.cement-based surfaces (92%)  2.various other (8%) | 934 | 2.15 [1] (0.143) |
| Pheromone dispenser to insecticide distance (m) (distcat) | 1. ≤0.5m (42%)  2. >0.5≤1m (23%)  3. >1≤2m (21%)  4. >2m (15%) | 610 | 1.28 [3] (0.734) |

^1^ category 1 is the referent class
