# Supplementary figures and images for: Community deployment of a synthetic pheromone of the sand fly Lutzomyia longipalpis co-located with insecticide reduces vector abundance in treated and neighbouring untreated houses: Implications for control of Leishmania infantum
Source: PLoS Negl Trop Dis. 2021 Feb 3;15(2):e0009080. doi: 10.1371/journal.pntd.0009080 (PMC7886189; doi:10.1371/journal.pntd.0009080)

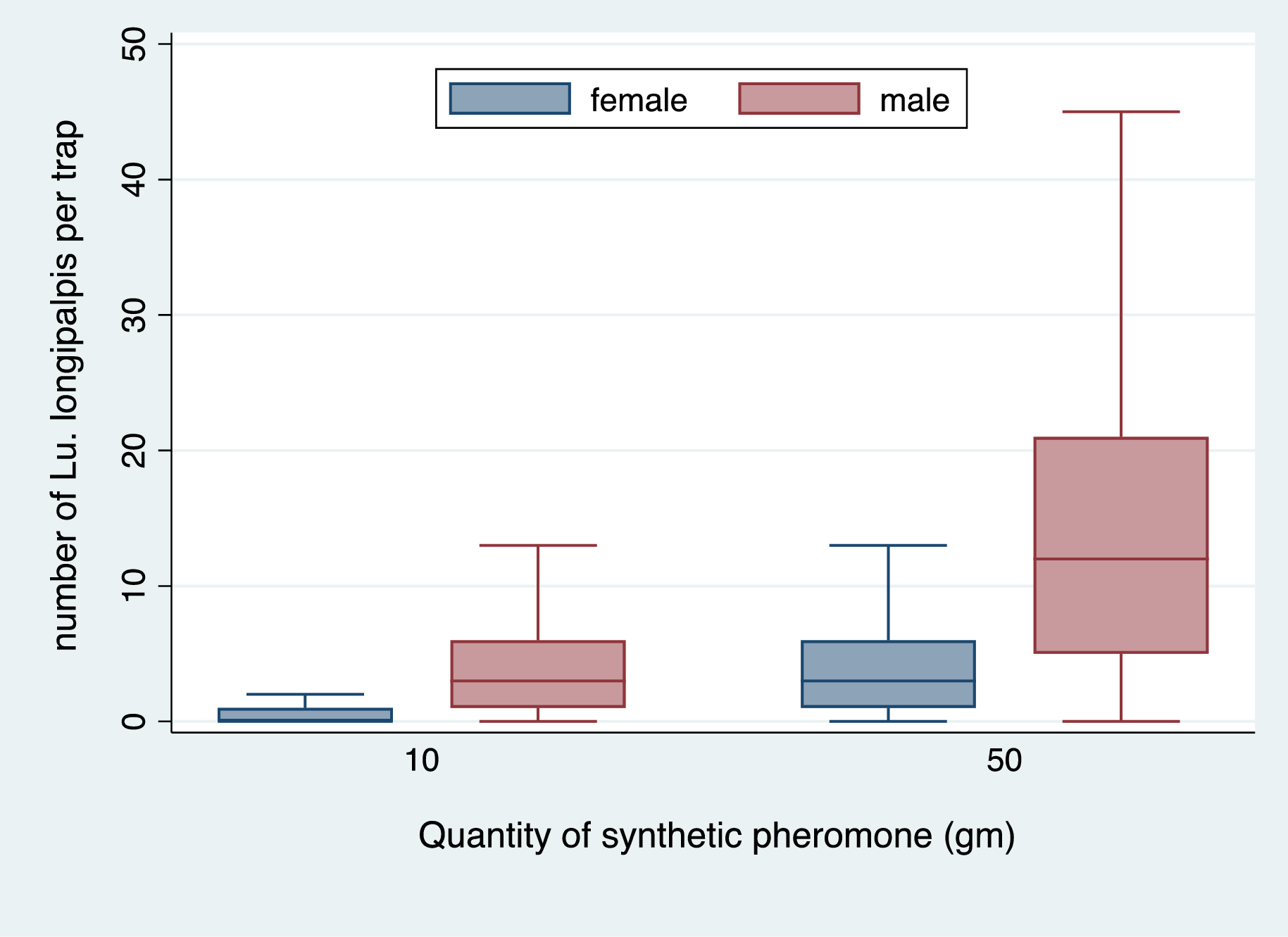

Supplement: S1 Fig — For graphical clarity data outliers are excluded. (TIF) [file pntd.0009080.s006.tif]

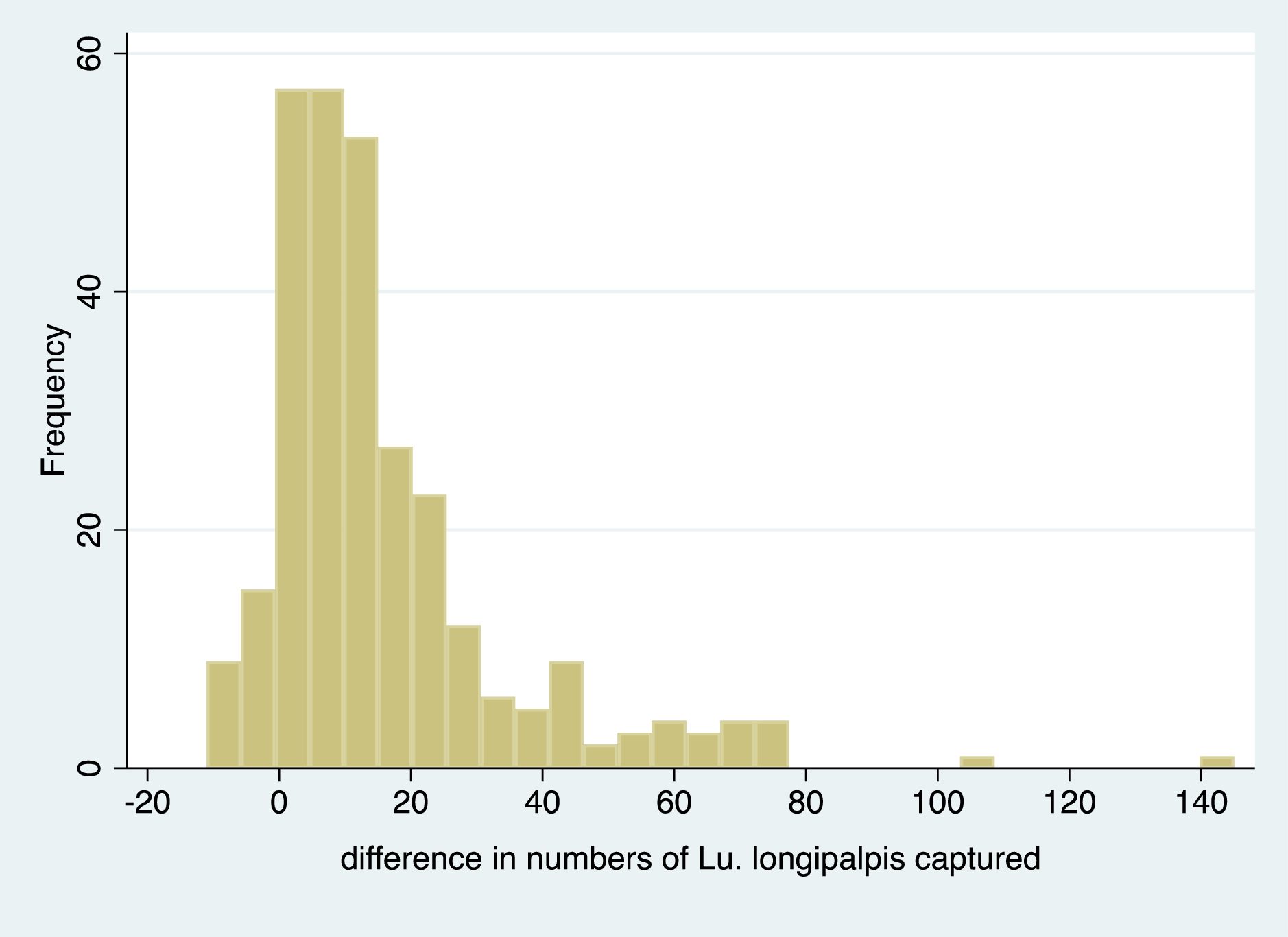

Supplement: S2 Fig — Data shown for sexes combined. (TIF) [file pntd.0009080.s007.tif]

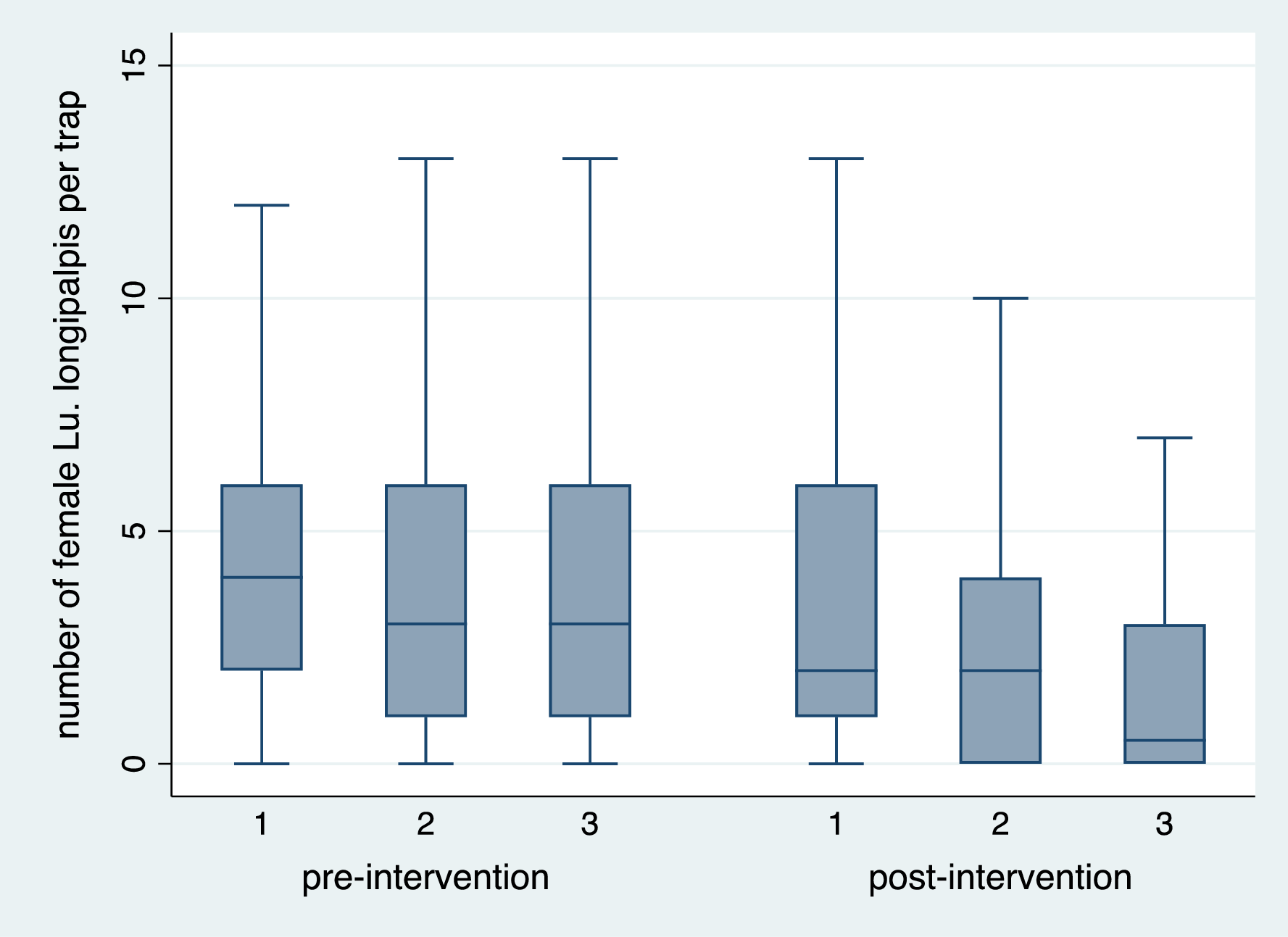

Supplement: S3 Fig — For graphical clarity data outliers are excluded. (TIF) [file pntd.0009080.s008.tif]

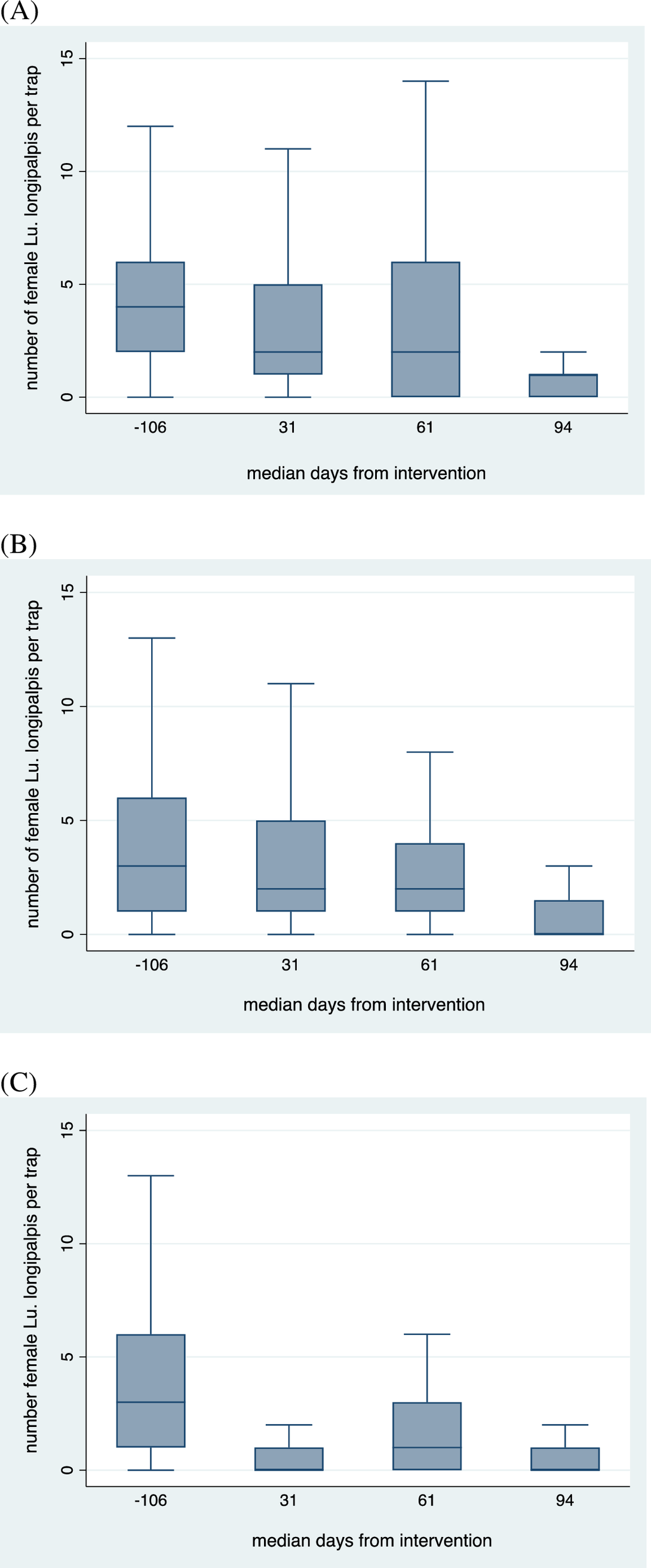

Supplement: S4 Fig — Data shown for sample rounds 1–4 represented by the median days from intervention. For graphical clarity data outliers are excluded. (TIF) [file pntd.0009080.s009.tif]

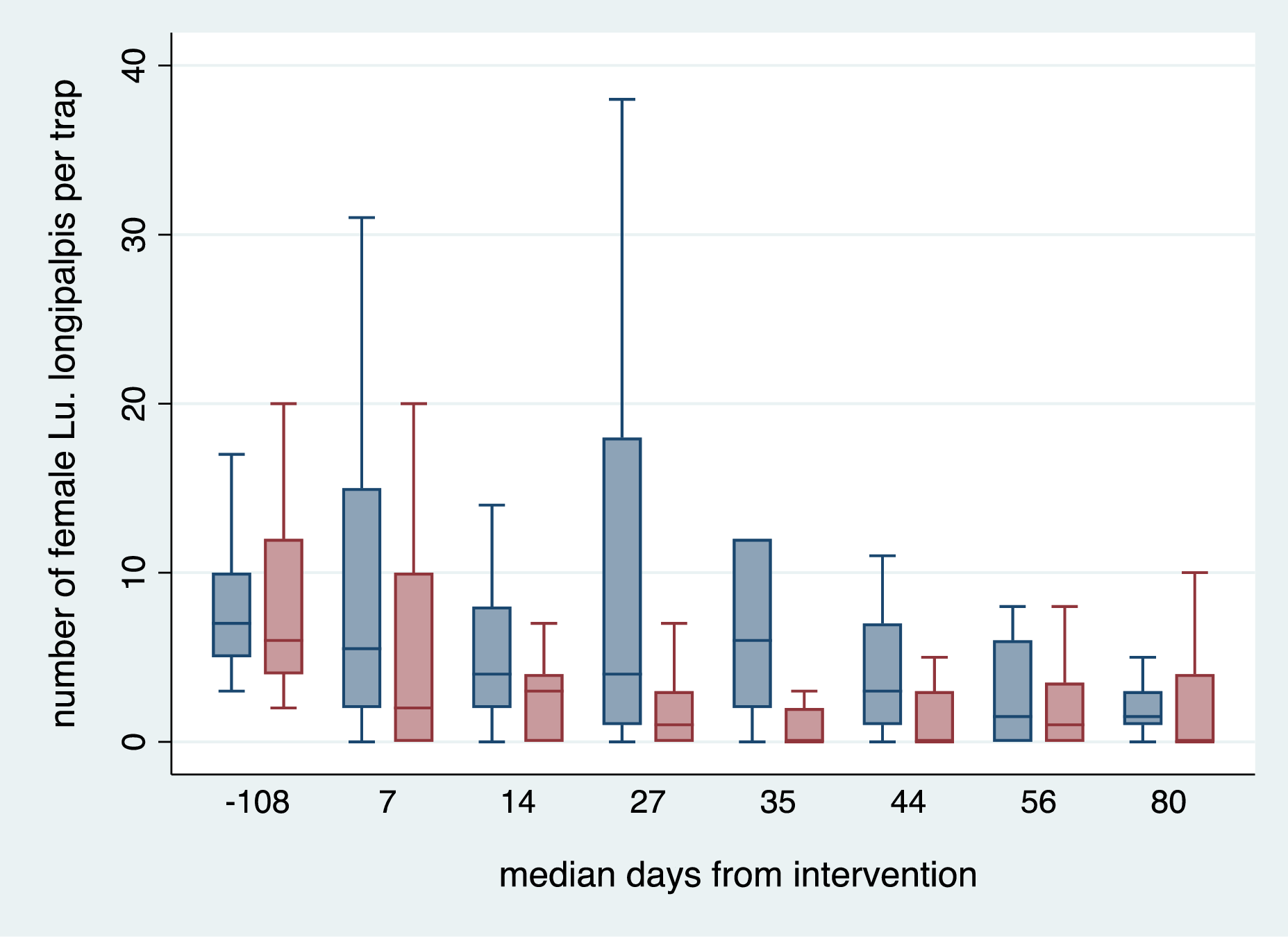

Supplement: S5 Fig — Data shown for each sample round represented by the median days from intervention. (TIF) [file pntd.0009080.s010.tif]

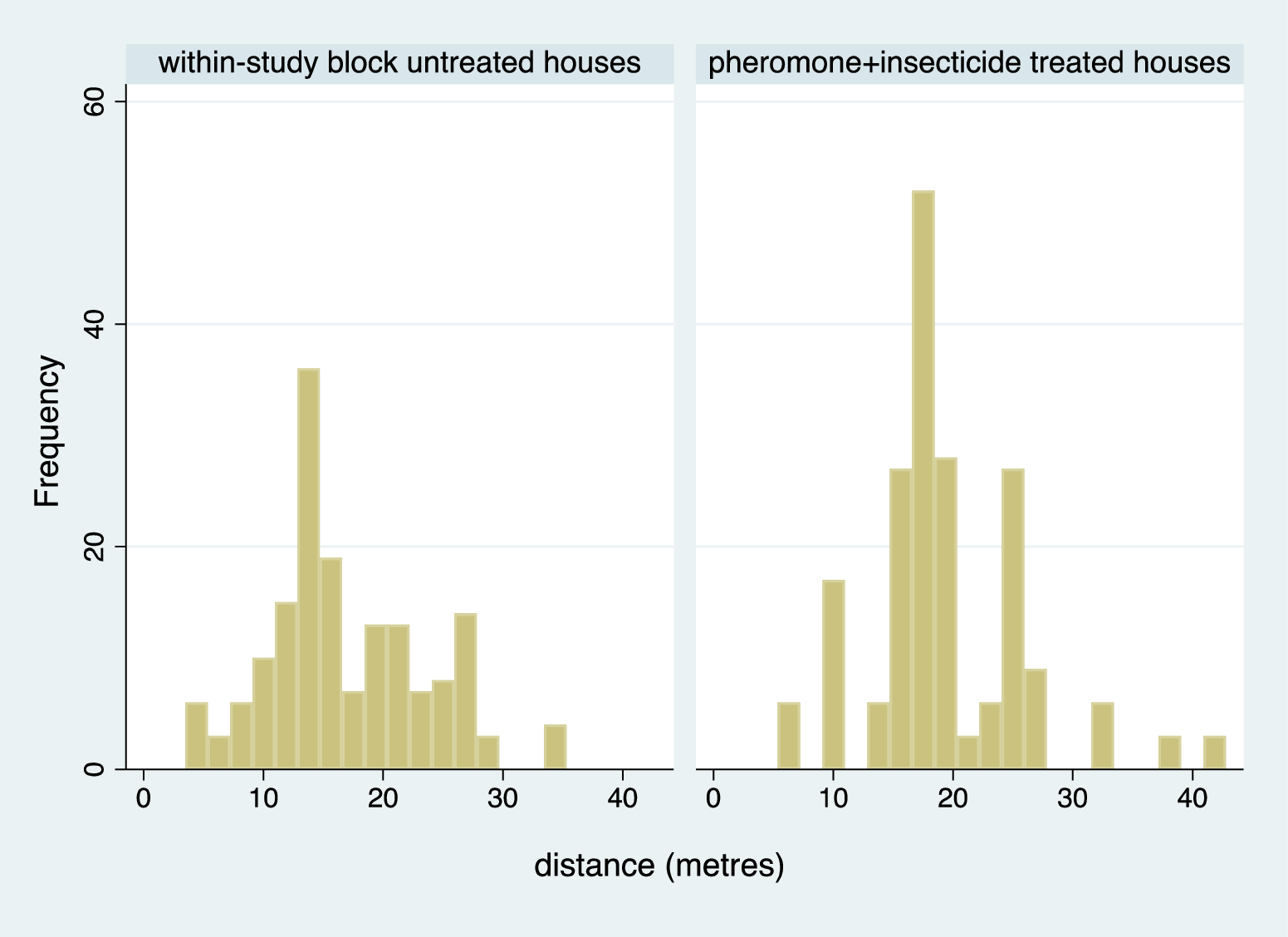

Supplement: S6 Fig — (TIF) [file pntd.0009080.s011.tif]

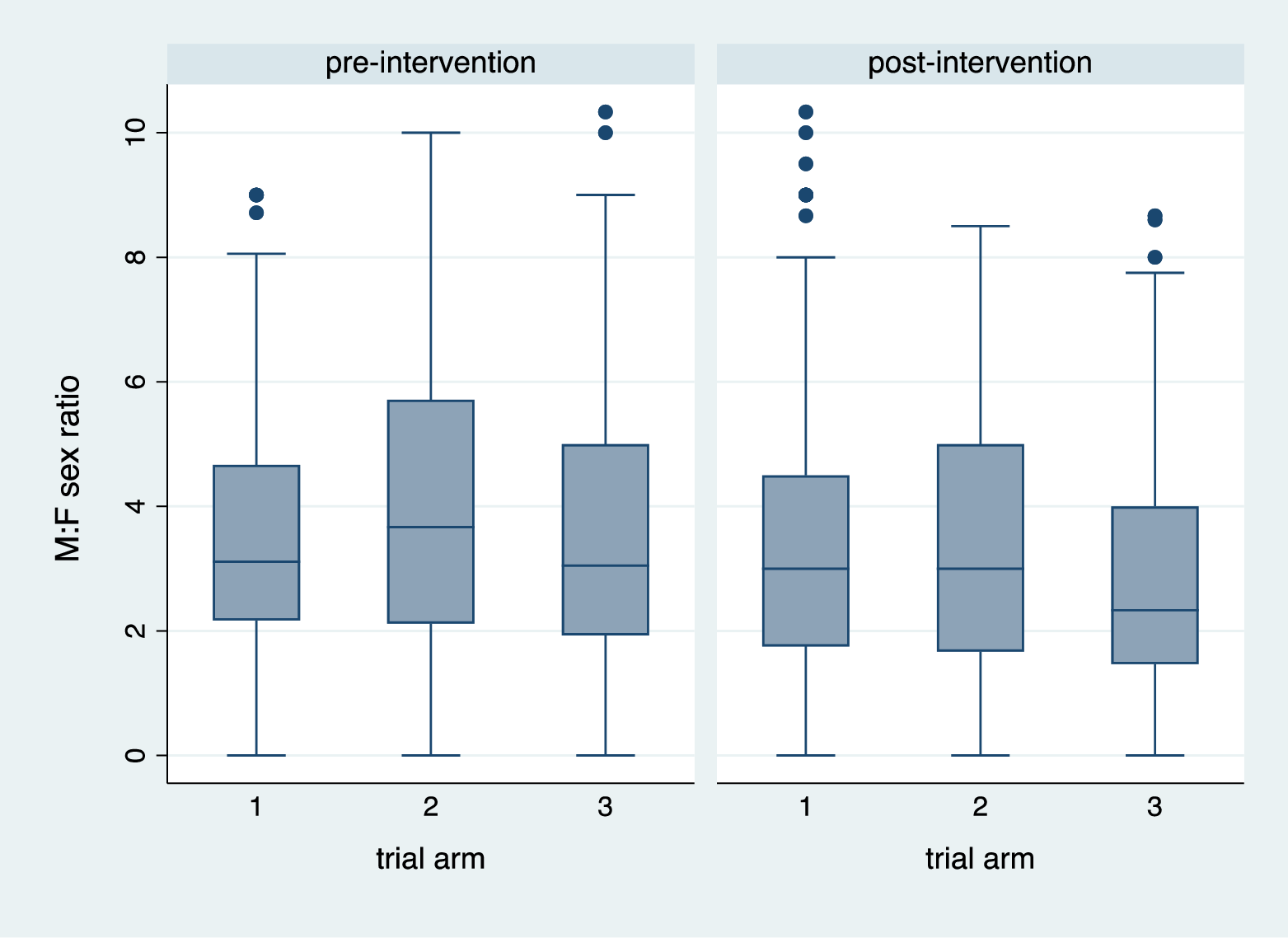

Supplement: S7 Fig — For graphical clarity data outliers are excluded. (TIF) [file pntd.0009080.s012.tif]
