# Supplementary material for: Community deployment of a synthetic pheromone of the sand fly Lutzomyia longipalpis co-located with insecticide reduces vector abundance in treated and neighbouring untreated houses: Implications for control of Leishmania infantum
Source: PLoS Negl Trop Dis. 2021 Feb 3;15(2):e0009080. doi: 10.1371/journal.pntd.0009080 (PMC7886189; doi:10.1371/journal.pntd.0009080)
Supplement: Attachment — Submitted filename: Saini comments on PLOS Sandfly MS 28.10.20.docx [file pntd.0009080.s013.docx]

This is an interesting study wherein authors have investigated the use of ‘lure’ (synthetic male sex aggregation pheromone) and ‘kill’ (insecticide) to investigate whether this strategy can reduce vector numbers. In the process they find that their treatments had spatial effects on neighboring households in term of vector reduction. This is important work for developing integrated approaches to control these vectors of visceral leishmaniasis. It is also interesting that very high doses of the pheromone can have spatial effects.

Authors have clearly missed the opportunity to include more treatments in this study which could have clearly defined the role of pheromone alone, insecticide alone or kairomones from chicken or dogs in various combinations to make concrete conclusions for future integrated control interventions and a better manuscript. There are several other points listed below which authors need to address.

1. Title: is misleading ‘*protection* is usually associated with *reduction* in disease levels. In their study disease incidence was not measured. Only the effect of the pheromone + insecticide target on vector abundance was investigated.
2. Lines 29-31 Authors should clearly state the objective of their study. Objective is not clear. What do they mean by “spatial efficacy”? Primary objective was to determine the effect of the “lure” (pheromone) & “kill” (insecticide target) technique on reducing sand fly populations and eventually reduce disease incidence.
3. “Community intervention”- authors loosely mention at many places “**community intervention”** (e.g. first page of their MS and Line 44 of Abstract) yet in the entire manuscript they do not describe clearly what the role of the community was in the trial.
4. **Treatments:** Abstract lines 32-40 & Treatment Interventions. Basically authors had only two treatments:

Pheromone+insecticide target and placebo or true control. Distinction between placebo and true control is not easy to understand. See also line 257.

In their treatment plan why did the authors not include in their study, amongst their treatments: insecticide target alone, pheromone treatment alone (could be with a sticky target or CDC trap). In this way the effect of the pheromone or insecticide treatments alone or in combination could be determined! These additional treatments would have assisted to make better conclusions.

5. Lines 37-40: why were fly numbers caught in CDC traps monitored monthly while fly numbers in sentinel houses with pheromone +insecticide monitored every 7-9 days’ intervals?
6. Line 45: female fly numbers were reduced by 59% also mentioned in the results. This reduction is with respect to baseline entomological data (pre-intervention data)?
7. Lines 49-50: 24% protection against female vectors is calculated how? This is reduction in disease incidence or merely fly numbers?
8. Line 52: what are intervention effects?
9. Lines 80-82: there is definitely a major reduction in amount of insecticide used compared to IRS for averaged sized houses? Authors have mentioned in the MS the cost of the pheromone is < US$1per unit. How many units were used to achieve the current results? What about the cost of dispensers? Of course how long will the insecticide treatment last on a target in the current study or provide protection in the entire house or how long will the pheromone last in the dispensers are amongst many other considerations which must be taken into account.
10. Introduction: what is the current VL disease level in Brazil?
11. Lines 117-120: a little bit more info on the publication referred to is needed e.g. were the treatments the same as in the current study? What was the reduction in fly catches per trap per day? and what was the disease reduction in canine hosts? This study is apparently the pre-cursor of the current study and it would benefit readers tremendously if the result were mentioned in a little bit of detail.
12. Lines 122-123: authors must describe what do they mean by “spatial community effect”. Spatial effect of the pheromone can be understood but combination with community? Reduction in fly numbers and effect on disease levels in people??
13. Lines 129-132: “households with and without non-human hosts”? By none-humans you mean with dogs or chicken? Can a house hold be there without humans?
14. Lines 134-135: main objective was to **quantify community effects of the treatments**? See also comment 3 above.
15. Study design line 169: “treatment as detailed below”. Then again on line 169 authors say again as detailed below. Why don’t authors straight away outline the treatments clearly and then indicate how these were allocated randomly to various blocks instead of creating suspense for the reader?!! See also comment 4 above.
16. Line 173 ‘plum’ is ‘plume’
17. Pheromone intervention: line 207 onwards: Did the authors measure the weight of the dispensers before and after treatment to determine the dose released from them per day? That would give the authors indication on how much quantity to use over time and design better dispensers.
18. Line 271: *“lost- to- follow- up”* Not clear!
19. Table 1 lines 281: Please clarify what TC, PC IRS stand for? Table legend is not clear. Tables should be self-explanatory.
20. Lines 405-409: Please indicate how many flies/trap/day were you catching in the various treatment blocks pre-intervention.
21. Line 436: what is duration of community effect? Not clear. See also previous comments in this respect.
22. Line 450: protective effect? In terms of disease level or fly numbers?
23. Discussion: first 2 paragraphs may be shifted to Introduction Lines 540 to 562
24. Lines 600-601: Not clear 2 to 3 months?
25. Lines 603 – 607: Some clarification is needed. Authors previously indicate 50 mg pheromone lures were optimal for current study (line 401-402). Now they want to recommend 10 mg lures as per previous CRT? Please clarify.
26. **Role of chickens in attraction of sand flies in current study:** Lines 626 to 644 comprise of contradicting statements. Authors should clarify clearly what this study or the previous studies indicate on the role of non-human hosts (dogs or chicken) in host attraction of sand flies. This is important to guide future interventions.

These non-human hosts definitely play a major role in the epidemiology of the disease and in any integrated interventions kairomones emanating from these can be exploited to enhance suppression of vector levels and thus impact disease levels. I would urge authors to read some of the tsetse control papers where insecticide targets and host kairomones have been used for suppression of vectors.

**See below authors statements:**

629 [14, 15, 53]. The results of the present study confirm the synergism of chicken odour and the

630 synthetic pheromones on *Lu. longipalpis* attraction. Vector numbers captured at pre

631 intervention households were positively associated with larger numbers of chickens, and the

632 presence *versus* absence of household chickens disproportionately influenced the intervention

633 outcomes, resulting in a 1.65 and 1.46 times increase in protective response against female

634 *Lu. longipalpis* in pheromone-treated and within-block placebo houses, respectively.

639 dog sleeping sites/kennels where CDC traps were set in parallel [18]. We did not detect

640 changes in *Lu. longipalpis* numbers related to the abundance of other non-human hosts in that

641 study or in the current study, though correlations have been reported elsewhere [39].

642 Importantly, we show that a similar impact against the vector is achieved as in the previous

643 CRT, here by applying the intervention to an exterior wall, and which does not require the

644 presence of chicken or other animal hosts.

**So what is the way forward?**

If previous study (Lines 636-638) showed that 60% of the vectors are present at the chicken roosting sites, then in any intervention placing an insecticide + pheromone target here would make sense. Why did the authors in the present study ignore this? What is the rationale behind this decision? Of course putting insecticide on a house wall is easier. Alternatively, authors can put insecticide treated screens like those used for tsetse flies. Even pheromone dispensers could be hanged on these screens. Some food for thought!

**Dr. R.K. Saini**

Principal Scientist & CEO

**Pesti*nix Ltd.***

**International Pest & Vector Specialists**

[rsaini@pestinix.com](mailto:rsaini@pestinix.com)
