# Supplementary material for: Community deployment of a synthetic pheromone of the sand fly Lutzomyia longipalpis co-located with insecticide reduces vector abundance in treated and neighbouring untreated houses: Implications for control of Leishmania infantum
Source: PLoS Negl Trop Dis. 2021 Feb 3;15(2):e0009080. doi: 10.1371/journal.pntd.0009080 (PMC7886189; doi:10.1371/journal.pntd.0009080)
Supplement: Attachment — Submitted filename: Response to reviewers 11-11-20 .docx [file pntd.0009080.s014.docx]

Dear Editor,

We thanks the reviewers for their helpful suggestions. Our responses to their queries are outlined below. Consequently, we have substantially rephrased or rearranged parts of the manuscript and clarified some terminology.

**Reviewer #2**

(i) In the Introduction, include a brief highlight of the previous study by Courtenay et al (2019, PLOS NTD) and rationale underlying the present study

>>the Introduction has been amended to more clearly provide the background and rationale to this study (as suggested also by Reviewer 3)

(ii) in the Discussion, include the need to identify the kairomonal blend(s) associated with chicken and dogs, and their potential for deployment with the pheromone.

>> We have added a statement in the Discussion that future work could address this possibility, however it is a different set of studies requiring identification and synthesis of host odours and field trials, and not really our concern in this paper (see also our next response).

**Reviewer #3**

Authors have clearly missed the opportunity to include more treatments in this study which could have clearly defined the role of pheromone alone, insecticide alone or kairomones from chicken or dogs in various combinations to make concrete conclusions for future integrated control interventions and a better manuscript.

>> The method under scrutiny in this study is the “lure-and-kill” package, thus the objective is not to differentiate the effects of the two components, as it would no longer represent a novel vector control tool. We agree that there are many potential treatments that could be tested but requiring substantial additional resources to power such as multi-arm study. Testing “kairomones from chicken or dogs in various combinations” is currently not possible as they first require identification and synthesis. The rationale for *this study* is now more clearly outlined in the Introduction.

1. Title: is misleading ‘*protection* is usually associated with *reduction* in disease levels. In their study disease incidence was not measured. Only the effect of the pheromone + insecticide target on vector abundance was investigated.

>>We have altered the title to be more explicit. Similarly, we have made alterations throughout the text to replace protection with reduction, but at the same time we use the term protection in places in the text based on the argument that reducing the abundance of the vector in houses is synonymous with providing protection against vectors in houses.

2. Lines 29-31 Authors should clearly state the objective of their study. Objective is not clear. What do they mean by “spatial efficacy”? Primary objective was to determine the effect of the “lure” (pheromone) & “kill” (insecticide target) technique on reducing sand fly populations and eventually reduce disease incidence.

>>the sentence in the Abstract has been altered for clarity, to “..tested for the intervention effects on vector abundance, hence potential transmission,..”.

3. “Community intervention”- authors loosely mention at many places “community intervention” (e.g. first page of their MS and Line 44 of Abstract) yet in the entire manuscript they do not describe clearly what the role of the community was in the trial.

>>the term “community intervention” is used to describe what it is- a community-based intervention study, in the same sense that a cluster randomized trial is a community intervention trial, where the “role” of the community is that all eligible households or individuals within blocks / clusters are potentially recruited. We do not feel that there is need to change this term, however in the text we have made some alterations where perhaps previously it was not crystal clear.

4. **Treatments:** Abstract lines 32-40 & Treatment Interventions. Basically authors had only two treatments: Pheromone+insecticide target and placebo or true control. Distinction between placebo and true control is not easy to understand. See also line 257.

>>the referee is correct, there were only two treatments, but three trial arms, so somewhat confusing. As the study design dictated, we needed to differentiate the two groups of placebo houses, which for convenience we labelled as “treatments” We have now made the distinction clear and highlighted this also in the trial design section. We now label the placebo groups as “Untreated” and “True Control” houses throughout the text, and in figure and table legends.

5. Lines 37-40: why were fly numbers caught in CDC traps monitored monthly while fly numbers in sentinel houses with pheromone +insecticide monitored every 7-9 days’ intervals?

>>As already stated in the Methods, the smaller number of sentinel houses were monitored more frequently to detect changes in sand fly numbers at finer temporal resolution, and which to complement the more cross-sectional nature of the monthly follow-up of the total recruited houses. Logistical restraints prevented all houses being monitored at short intervals.

6. Line 45: female fly numbers were reduced by 59% also mentioned in the results. This reduction is with respect to baseline entomological data (pre-intervention data)?

>>Under the Statistical Analysis section in the Methods, we state that “The numbers of *Lu. longipalpis* captured per house trap night in Pheromone and Untreated arms were each compared to catch numbers in the True Control arm”. This is not an analysis of pre- *versus* post-intervention within treatment group, as the numbers of sand flies could naturally vary over time e.g. due to climatic conditions. However, in the same section, we state that “Models included interaction terms: arm × pre-/post-intervention …as appropriate”. This essentially controls for the baseline values in all groups.

7. Lines 49-50: 24% protection against female vectors is calculated how? This is reduction in disease incidence or merely fly numbers?

>> This value, as all statistical model outcomes, are calculated by standard epidemiological procedures: y= 1- incidence risk ratio (IRR).

8. Line 52: what are intervention effects?

>> this is a standard statistical/epidemiological term which refers to the intervention “effect” based on the model coefficients, as already stated in the Statistical Analyses section. Intervention studies use this term regularly. We do not feel that this is ambiguous, certainly no more than “outcome measure” being less cumbersome than “changes in the dependent variable relative to independent variable”.

9. Lines 80-82: there is definitely a major reduction in amount of insecticide used compared to IRS for averaged sized houses?

>>There is no doubt that the amount of insecticide used here to cover a 2.6m^2^ surface area was substantially less compared to covering an entire house of 250m^2^, typical of IRS campaigns.

Authors have mentioned in the MS the cost of the pheromone is < US$1per unit. How many units were used to achieve the current results? What about the cost of dispensers? Of course how long will the insecticide treatment last on a target in the current study or provide protection in the entire house or how long will the pheromone last in the dispensers are amongst many other considerations which must be taken into account.

>> we have added to the Discussion the number of houses treated with lures and protected (the numbers of lures used per houses is already in the Methods). The costs of the dispensers are negligible and now stated in the text form part of the quoted pheromone costs. In the Discussion, we have also referenced a modelling study based on these and CRT data of the estimated unit costs of the synthetic pheromone relative to different implementation scenarios and efficacies against human infection. And we added a sentence to reflect other variables that need to be considered in estimating the final costs.

10. Introduction: what is the current VL disease level in Brazil?

>>We have added infection estimates for Brazil to the Introduction

11. Lines 117-120: a little bit more info on the publication referred to is needed e.g. were the treatments the same as in the current study?

>>the background and rationale of the study are now rewritten in the Introduction and the Discussion, i.e. how the two studies differed and why we designed the current study as we did. We also include statements about the amounts of pheromone used (10mg versus 50mg) and the different target location in each study, in the Methods, Results and Discussion.

What was the reduction in fly catches per trap per day? and what was the disease reduction in canine hosts? This study is apparently the pre-cursor of the current study and it would benefit readers tremendously if the result were mentioned in a little bit of detail.

>>We have added more detail of the previous CRT study to the text in both the Introduction and Discussion, included the % reductions in canine infection incidence. We have carefully considered the suggestion to provide % reductions in vector numbers per day. We are very concerned about assuming a liner relationship to convert the changes in numbers over the observed periods into a reduction per day, as we feel this would be misleading, or at worst incorrect. Even more inaccurate would be a comparison between this and CRT study: the relationships between changes in vector abundance and time from intervention is likely to be non-linear, as indicated by predictive spatial models of this intervention’s effectiveness against canine and human VL incidence (Retkute 2020). Also, the study designs were very different (with different objectives) including not least the house recruitment criteria, the sand fly monitoring interval (monthly *versus* one follow-up after multiple replacement of the intervention over 24-30m), and the target location for synthetic pheromone and insecticide. Hence with these considerable caveats, we do not feel comfortable to include daily rates.

12. Lines 122-123: authors must describe what do they mean by “spatial community effect”. Spatial effect of the pheromone can be understood but combination with community? Reduction in fly numbers and effect on disease levels in people??

>>This has been altered throughout the text for clarity (also see responses above).

13. Lines 129-132: “households with and without non-human hosts”? By none-humans you mean with dogs or chicken? Can a household be there without humans?

>>This has been altered in the Introduction text for clarity.

14. Lines 134-135: main objective was to quantify community effects of the treatments?

>>This has been altered in the Introduction text for clarity.

15. Study design line 169: “treatment as detailed below”. Then again on line 169 authors say again as detailed below. Why don’t authors straight away outline the treatments clearly and then indicate how these were allocated randomly to various blocks instead of creating suspense for the reader?!! See also comment 4 above.

>>This order is created because of the natural progression of the methodological procedures: recruitment, randomization, then intervention. This is our preference. If we change the order we will equally need to refer the reader to “below” for the reordered information.

However, if the editor feels that the order should be changed then we are willing to do so.

16. Line 173 ‘plum’ is ‘plume’

>>the plum has been altered

17. Pheromone intervention: line 207 onwards: Did the authors measure the weight of the dispensers before and after treatment to determine the dose released from them per day? That would give the authors indication on how much quantity to use over time and design better dispensers.

We did not measure the weight loss over time in the field because this would not be an accurate representation of pheromone release - the dispensers become contaminated with dirt and mold and weight would not be solely related to pheromone release, will fluctuate with ambient temperature and other environmental conditions (humidity, wind speed, etc). However, we acknowledge that a fully specified controlled release device would be advantageous. In a previous study (Bray et al 2014), we measured the activity of the dispenser over time, and have already cited this in the manuscript.

18. Line 271: *“lost- to- follow- up”* Not clear!

>>the term lost-to-follow-up is a standard term for any cohort or intervention study. We do not feel that this term requires further elaboration.

19. Table 1 lines 281: Please clarify what TC, PC IRS stand for? Table legend is not clear. Tables should be self-explanatory.

>>we have altered the legends and checked all Tables and Figures to include explanation of these abbreviations and to render them self-explanatory (see also responses above).

20. Lines 405-409: Please indicate how many flies/trap/day were you catching in the various treatment blocks pre-intervention.

>>The geometric mean number of vectors captured per trap night per treatment arm pre- and post-intervention and per follow-up round are already shown in Supplementary Figures S3, S4, S5.

21. Line 436: what is duration of community effect? Not clear. See also previous comments in this respect.

>>We have clarified the terminology “duration of the intervention effect” in the Results and throughout the text for clarity (see responses above).

22. Line 450: protective effect? In terms of disease level or fly numbers?

>The term “protective effect” has been made more explicit, described as against female and male fly abundance, or altered to “reductions” throughout the text (see responses above).

23. Discussion: first 2 paragraphs may be shifted to Introduction Lines 540 to 562

>>This has been completed and elaborated

24. Lines 600-601: Not clear 2 to 3 months?

>>> The statement “for at least two months, but for less than three months” is because the interval of measurement (Figures 3 & 4) does not allow us to be more specific. We have added reference to Figures 3 & 4 after the statement in the Discussion to make clear.

25. Lines 603 – 607: Some clarification is needed. Authors previously indicate 50 mg pheromone lures were optimal for current study (line 401-402). Now they want to recommend 10 mg lures as per previous CRT? Please clarify.

>>>The reviewer’s query is not clear as in fact we are not recommending the use of 10mg in this paragraph, but simply reporting the results of the previous CRT. Then we comment on the replacement rate and that increasing the longevity of the controlled-release pheromone dispensers requires further work. Elsewhere in the text, we discuss the differences between the two study designs..

26. Role of chickens in attraction of sand flies in current study: Lines 626 to 644 comprise of contradicting statements. Authors should clarify clearly what this study or the previous studies indicate on the role of non-human hosts (dogs or chicken) in host attraction of sand flies. This is important to guide future interventions.

>>We agree that this section was confusing, so we have now separated out the two issues and rewritten the text to make it clearer. We state that the pheromone intervention is enhanced by the presence vs absence of chickens but does not require chickens (odour) to be effective, and that there is apparent synergism between the pheromone and chicken odour. We also comment on the possible effects of chicken / host abundance on vector numbers, and the complex dynamics governing sand fly recruitment to leks.

639-641 We did not detect changes in *Lu. longipalpis* numbers related to the abundance of other non-human hosts in that study or in the current study, though correlations have been reported elsewhere [39].

>>This latter statement refers to **other hosts**, not to chickens, but has been made clear in relation to the above query.

These non-human hosts definitely play a major role in the epidemiology of the disease and in any integrated interventions kairomones emanating from these can be exploited to enhance suppression of vector levels and thus impact disease levels. I would urge authors to read some of the tsetse control papers where insecticide targets and host kairomones have been used for suppression of vectors.

>>Yes, this is an interesting idea, and we are aware of studies on attractants used for vector systems. However we are unaware of studies of host kairomones showing that “…can be exploited to enhance suppression of vector levels..” against VL vectors. As responded above, this requires substantial work, and not the subject of this study. The synthetic pheromone is the lure tested in this study, and is shown to attract greater number of sand flies that natural pheromone with or without the presence of hosts. We comment in the Discussion on the possibility to conduct future studies of host kairomones.

**So what is the way forward?**

If previous study (Lines 636-638) showed that 60% of the vectors are present at the chicken roosting sites, then in any intervention placing an insecticide + pheromone target here would make sense. Why did the authors in the present study ignore this? What is the rationale behind this decision? Of course putting insecticide on a house wall is easier. Alternatively, authors can put insecticide treated screens like those used for tsetse flies. Even pheromone dispensers could be hanged on these screens. Some food for thought!

>>Indeed, food for thought. The purpose of the current study was to compliment the previous trial and, as stated in the rationale, to test the intervention on the mixed community where not all houses own chickens, or other animals. Hence, for future public health deployment, this was one important reason why the current study was designed in this way.

As a side note, the Brazilian Ministry of Health (represented by one co-author) are engaged and were involved in setting the objectives of this study.

Ethical statement

We have added / clarified in the Ethical Considerations section that no clinical samples or personalized human data were collected, that fully informed written consent was obtained, and that the study protocols were approved by the local Ministry of Health.
